# Langerhans Cell Histiocytosis of the Thyroid Leading to the Diagnosis of a Disseminated Form

**DOI:** 10.1155/2020/6284764

**Published:** 2020-03-17

**Authors:** Ibtissem Ben Nacef, Sabrine Mekni, Chedi Mhedhebi, Ines Riahi, Imen Rojbi, Mchirgui Nadia, Karima Khiari

**Affiliations:** ^1^Department of Endocrinology, Charles Nicolle Hospital, Tunis, Tunisia; ^2^Department of Otorhinolaryngology-Head and Neck Surgery, Charles Nicolle Hospital, Tunis, Tunisia

## Abstract

Langerhans cell histiocytosis (LCH) is a rare sporadic proliferative disorder of Langerhans cells. LCH rarely involves the thyroid gland. We report herein a case of a disseminated chronic form of LCH with a diagnosis established by histological examination of the thyroid gland. It is about a 37-year-old female who underwent total thyroidectomy for a thyroid nodule of the right lobe. Histological study showed a granulomatous thyroiditis, and the immunohistochemistry study revealed a strong positivity of histiocytes for the CD1 antigen and for the S100 protein. The incidence of LCH involving the thyroid gland, either as an isolated lesion or as a part of multisystemic disease, is extremely rare.

## 1. Introduction

Langerhans cell histiocytosis (LCH) is a rare sporadic proliferative disorder of Langerhans cells. It can cause either localized or generalized lesions, leading to the destruction of hard and soft tissues [1]. LCH rarely involves the thyroid gland. We report a case of a disseminated chronic form of LCH with a diagnosis established by histological examination of the thyroid gland.

## 2. Case Report

A 37-year-old female underwent surgery for a thyroid nodule of the right lobe. The nodule presented as suspicious because of its hard consistency, its adhesion to surrounding tissues, and the presence of calcifications on ultrasound. The patient was euthyroid before surgery. She had a total thyroidectomy. Histological study showed a granulomatous thyroiditis. The granulomas were composed of lymphocytes and histiocytes grouped into clusters. After surgery, the patient was referred to our establishment for investigation of a polyuropolydipsic syndrome and a one-year history of amenorrhea-galactorrhea.

Physical examination was normal except for bilateral galactorrhea. Urine specific gravity before test was 1.005. The water deprivation test results were in favor of a central diabetes insipidus (DI) (Table 1). Hormonal tests revealed pan-hypopituitarism, with a very low follicle-stimulating hormone (FSH) level of 0.9 mIU/mL (reference range 3.9–8.9 mIU/mL) and a luteinizing hormone (LH) level below 0.1 mIU/mL (reference range 2.1–10.9 mIU/mL). Baseline serum cortisol was 240 nmol/L and did not respond to insulin hypoglycemia test. An inappropriate adrenocorticotropic hormone (ACTH) level of 12 pg/mL suggested secondary adrenal insufficiency. Likewise, baseline GH was 0.25 ng/mL and did not respond to insulin hypoglycemia test. Prolactin level was mildly elevated (2520 mIU/mL with a reference range of 71–567 mIU/mL), which was consistent with disconnection hyperprolactinemia. As for the thyroid function tests, they were noninterpretable because the patient had already been on therapy. However, it is important to note that thyroid-stimulating hormone (TSH) level responded to TRH stimulating test. MRI of the hypothalamic-pituitary axis showed an infiltrative process of the hypothalamus with a loss of the posterior pituitary bright spot. The patient was discharged; she received hormone replacement therapy including desmopressin acetate 20 µg intranasal solution per day, oral hydrocortisone 20 mg per day, and levothyroxine 150 *μ*g per day.

**Figure 1:**
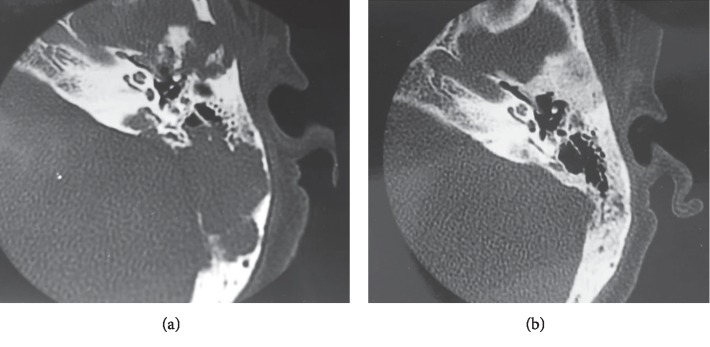
Scan of the left temporal bone in axial section, bone window. (a) Extensive lytic lesions (arrow). (b) Complete regression of the lytic lesions after treatment.

**Figure 2:**
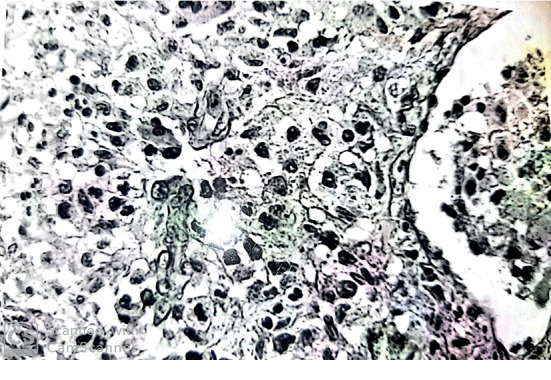
Granulomas of tumor histiocytes with an oval nucleus centered by a small nucleolus; their cytoplasm is weakly eosinophilic.

Eight months later, she presented with a left retroauricular bone pain and preauricular and submandibular lymphadenopathies, along with dental pain and tooth mobility. The facial and temporal bones CT-scan revealed a lytic lesion of the horizontal branch of the right mandible and a significant lytic lesion of the left petrous bone, extended to the squama (Figure 1(a)). The diagnosis of LCH was then strongly suspected, which led to re-examination of the excised thyroid gland by the pathology department. The immunohistochemistry study was performed, and it revealed a strong positivity of histiocytes for the CD1 antigen and the S100 protein (Figure 2).

The extension assessment of the disease included a thoracic CT-scan that revealed peripheral pulmonary micronodules, some of which were excavated. The treatment consisted of a chemotherapy combining vinblastine at a dose of 5 mg weekly and prednisone at a dose of 0.5 mg/kg daily. After four months of treatment, favorable evolution was noted, with a good tolerance of treatment, a clear relief of the headache, and disappearance of the left retroauricular pain. The control temporal bones CT-scan showed complete regression of the lytic lesion (Figure 1(b)). Nevertheless, the patient still required the same doses of pituitary hormones replacement therapy.

## 3. Discussion

Langerhans cell histiocytosis is a rare disease that affects predominantly, but not exclusively, children. It has an incidence of up to 9 cases per million children in Western Europe, dropping to 1-2 cases per million in adults [2]. LCH of the adult preferably affects the bones, lungs, skin, and posterior pituitary gland. The most common endocrinological manifestation of LCH is diabetes insipidus, which is a consequence of posterior pituitary involvement. However, other manifestations such as anterior pituitary deficiency are observed, resulting in secondary or tertiary hypothyroidism [3].

Our patient presented with DI and amenorrhea-galactorrhea syndrome as the clinical feature of disconnection hypothalamic hyperprolactinemia, which is caused by the histiocytic infiltration of the pituitary hypothalamus including stalk.

Pan-hypopituitarism, as in our case, is rare. Pituitary insufficiency is explained by the lack of secretion of hormones from the hypothalamus and pituitary [4].

The incidence of LCH involving the thyroid gland, either as an isolated lesion or as a part of a multisystemic disease, is extremely rare. Thyroid LCH is much more common in adults than in children, especially for females [5–8].

Patten et al. [9] reviewed 66 cases of LCH with thyroid involvement and observed an overall median age of 28 years with the majority of the patients presenting clinically with euthyroid goiter. Of the 66 cases, solitary thyroid involvement was noted in only 17 cases (25.7%) with all, except one, presenting in adulthood. Our patient was 37 years old and presented a solitary thyroid involvement initially.

Hypothyroidism during LCH can be either primary or most commonly secondary. In our case, it was due to a TRH (thyrotropin-releasing hormone) secretion deficiency. The normal response to TRH stimulation test supports this assumption.

Clinically, thyrotropic insufficiency is distinguished from primary hypothyroidism by the absence of myxedema and mainly by the clinical features of other pituitary deficiencies, as it is usually part of an anterior pan-hypopituitarism.

Thyroid gland involvement leads in the majority of cases to confusion and diagnostic mistakes. Lahey et al. (out of 10) reported a case of LCH with thyroid involvement; the diagnosis of thyroiditis was initially suspected in light of the results of the fine needle aspiration (FNA). The diagnosis was corrected after thyroidectomy. El Halabi et al. [10] also reported a case of LCH with thyroid localization. The result of the FNA cytology was in favor of either a lymphocytic thyroiditis or papillary carcinoma. The diagnosis was confirmed after histological study of the thyroidectomy specimen. In our case, thyroid involvement appeared eight months before hypothalamic involvement manifestations, which was unknown.

The initial misdiagnosis of granulomatous thyroiditis is due to the inflammatory character of LCH. In fact, the formation of highly active inflammatory granuloma and the recruitment of additional inflammatory cells such as eosinophils, lymphocytes, and neutrophils are explained by the expression of proinflammatory cytokines in the mutated LCs. This led some authors to the definition of LCH as an *inflammatory myeloid neoplasia* [11].

To establish the diagnosis of LCH, it is required to have histopathological analyses identifying tissue infiltration by histiocytes with characteristics of LCs. Pathologic histiocytes in LCH are mononucleated cells with coffee bean- or kidney-shaped nuclei. Detection of LC markers is mandatory to confirm the diagnosis. In routine practice, detection of Birbeck granules by electron microscopy has been widely replaced by detection of CD1a and CD207 expression, whereas expression of protein S100 is not specific. LCH cells are often associated with abundant eosinophils and multinucleated giant cells [12]. In our case, the diagnosis of histiocytic infiltration of the thyroid was made retrospectively despite the presence of granulomas composed of lymphocytes, plasma cells, and especially histiocytes grouped into clusters. The immunohistochemical study of the thyroidectomy specimen was of a great contribution for the rectification of the diagnosis.

In conclusion, LCH is a rare disease, especially the thyroid gland involvement. The diagnosis is made through histological analysis and immunohistochemical staining. As in our case, the Langerhans cell histiocytosis of the thyroid can easily be missed and only established after typical manifestation of the disseminated form.

## Figures and Tables

**Table 1:** Water deprivation test results.

	Baseline	After water deprivation	After desmopressin
Diuresis (mL/h)	600	210	15
Urine specific gravity	1005	1010	1025
Serum osmolarity (mOsm/L)	303,4	319,4	286
Na (mEq/L)	147	154	138
